# A Neuronal Culture System to Detect Prion Synaptotoxicity

**DOI:** 10.1371/journal.ppat.1005623

**Published:** 2016-05-26

**Authors:** Cheng Fang, Thibaut Imberdis, Maria Carmen Garza, Holger Wille, David A. Harris

**Affiliations:** 1 Department of Biochemistry, Boston University School of Medicine, Boston, Massachusetts, United States of America; 2 Department of Biochemistry and Centre for Prions and Protein Folding Diseases, University of Alberta, Edmonton, Alberta, Canada; Creighton University, UNITED STATES

## Abstract

Synaptic pathology is an early feature of prion as well as other neurodegenerative diseases. Although the self-templating process by which prions propagate is well established, the mechanisms by which prions cause synaptotoxicity are poorly understood, due largely to the absence of experimentally tractable cell culture models. Here, we report that exposure of cultured hippocampal neurons to PrP^Sc^, the infectious isoform of the prion protein, results in rapid retraction of dendritic spines. This effect is entirely dependent on expression of the cellular prion protein, PrP^C^, by target neurons, and on the presence of a nine-amino acid, polybasic region at the N-terminus of the PrP^C^ molecule. Both protease-resistant and protease-sensitive forms of PrP^Sc^ cause dendritic loss. This system provides new insights into the mechanisms responsible for prion neurotoxicity, and it provides a platform for characterizing different pathogenic forms of PrP^Sc^ and testing potential therapeutic agents.

## Author Summary

Prion diseases are fatal neurodegenerative disorders that cause memory loss, impaired coordination, and abnormal movements. The molecular culprit in prion diseases is PrP^Sc^, an infectious isoform of a host-encoded glycoprotein (PrP^C^) that can propagate itself by a self-templating mechanism. Whether PrP^Sc^ itself is toxic to neurons, and if so, the cellular mechanisms by which it produces neuronal pathology are largely unknown, in part because of the absence of suitable cell culture models. We describe here a hippocampal neuronal cultural system to detect the toxic effect of PrP^Sc^ on dendritic spines, which are postsynaptic elements responsible for excitatory synaptic transmission, and which are implicated in learning, memory, and the earliest stages of neurodegenerative diseases. We found that purified, exogenously applied PrP^Sc^ causes acute retraction of dendritic spines, an effect that is entirely dependent on expression of PrP^C^ by target neurons, and on the on the presence of a nine-amino acid, polybasic region at the N-terminus of the PrP^C^ molecule. Both protease-resistant and protease-sensitive forms of PrP^Sc^ cause dendritic retraction. This system provides new insights into the mechanisms responsible for prion neurotoxicity, and it provides a platform for characterizing different pathogenic forms of PrP^Sc^ and testing potential therapeutic agents.

## Introduction

Prion diseases are fatal neurodegenerative conditions of humans and animals that have significantly impacted public health and the safety of the food and blood supplies. These disorders are caused by infectious proteins called prions, which propagate themselves by a self-templating mechanism in which PrP^Sc^, the infectious isoform, seeds conformational conversion of PrP^C^, a normal neuronal glycoprotein, into additional molecules of PrP^Sc^ [1–3]. Although this model for prion infectivity is now widely accepted, the cellular and molecular mechanisms by which prions actually cause neurodegeneration remain a mystery. There is a critical need to address this question in order to develop effective treatments for these currently incurable disorders.

The terminal neuropathology of prion diseases encompasses a number of features, including spongiform change, amyloid deposition, astrogliosis and neuronal loss[4]. However, some of the earliest and potentially most critical changes occur at the level of the synapse[5]. Synaptic pathologies, including loss as well as morphological and functional abnormalities of synapses, occur early during the course of many prion diseases, and PrP^Sc^ is often found in neuropil deposits that are referred to as “synaptic-like”, since they appear to surround synaptic sites[6–11]. Two-photon imaging studies of living, prion-infected animals demonstrate that swelling of dendritic shafts and retraction of dendritic spines occur early during the disease course, well before symptoms appear[12]. Taken together, these studies pinpoint synapses, in particular dendrites and dendritic spines, as important initial targets of prion neurotoxicity. Dendritic spines are protuberances on dendrites at which synaptic contacts (usually excitatory) occur[13]. Changes in their morphology are now believed to underlay synaptic plasticity associated with learning and memory, as well as degenerative events that occur during aging and neurological disease[14,15].

A major roadblock in studying prion neurotoxicity has been the lack of an experimentally tractable model system in which degenerative changes can be studied in cell culture. Having a neuronal culture system that is susceptible to the synaptotoxic effects of prions is crucial for delineating the underlying cellular and molecular mechanisms, assaying and characterizing different toxic species of PrP^Sc^, and potentially identifying drugs that block neurodegeneration. There are a limited number of cell lines capable of propagating prions in culture[16,17], and none of these exhibit signs of cytotoxicity as a result of chronic prion infection. Moreover, most of the cells used to propagate prions are transformed cell lines (e.g., N2a neuroblastoma cells), and there is very little published literature on prion infection of cultured primary neurons [18,19].

In this paper, we describe a new system that is capable of reproducing acute prion neurotoxicity, based on PrP^Sc^-induced degeneration of dendritic spines on cultured hippocampal neurons. The effects observed in our system are specific to PrP^Sc^-containing samples, require expression of full-length PrP^C^ by the target neurons, and are apparent within hours, well before chronic infection is established. Using this system, we have made several new observations relevant to prion neurotoxicity.

## Results

### Scrapie-infected brain homogenate is toxic to wild-type but not PrP-null neurons

We monitored the effect of PrP^Sc^-containing brain homogenates on the integrity of dendritic spines displayed by differentiated cultures of hippocampal neurons. Neurons in these cultures, which are maintained *in vitro* for 3 weeks in the presence of a feeder layer of astrocytes, develop morphologically mature axons and dendrites, and form functional excitatory and inhibitory synapses[20]. The dendrites are studded with a high density of mushroom-shaped and stubby spines (loci of glutamatergic, excitatory synapses), which can be stained with fluorescently labeled phalloidin by virtue of its ability to binds to actin filaments within the spines. After 24 hr of treatment with RML-infected brain homogenate (IBH) (final concentration of 0.16%) the neurons were fixed and stained with Alexa 488-phalloidin, and the number and area of spines were quantitated. The cultures were co-stained for tubulin to reveal the overall morphology of the dendrites.

**Fig 1:**
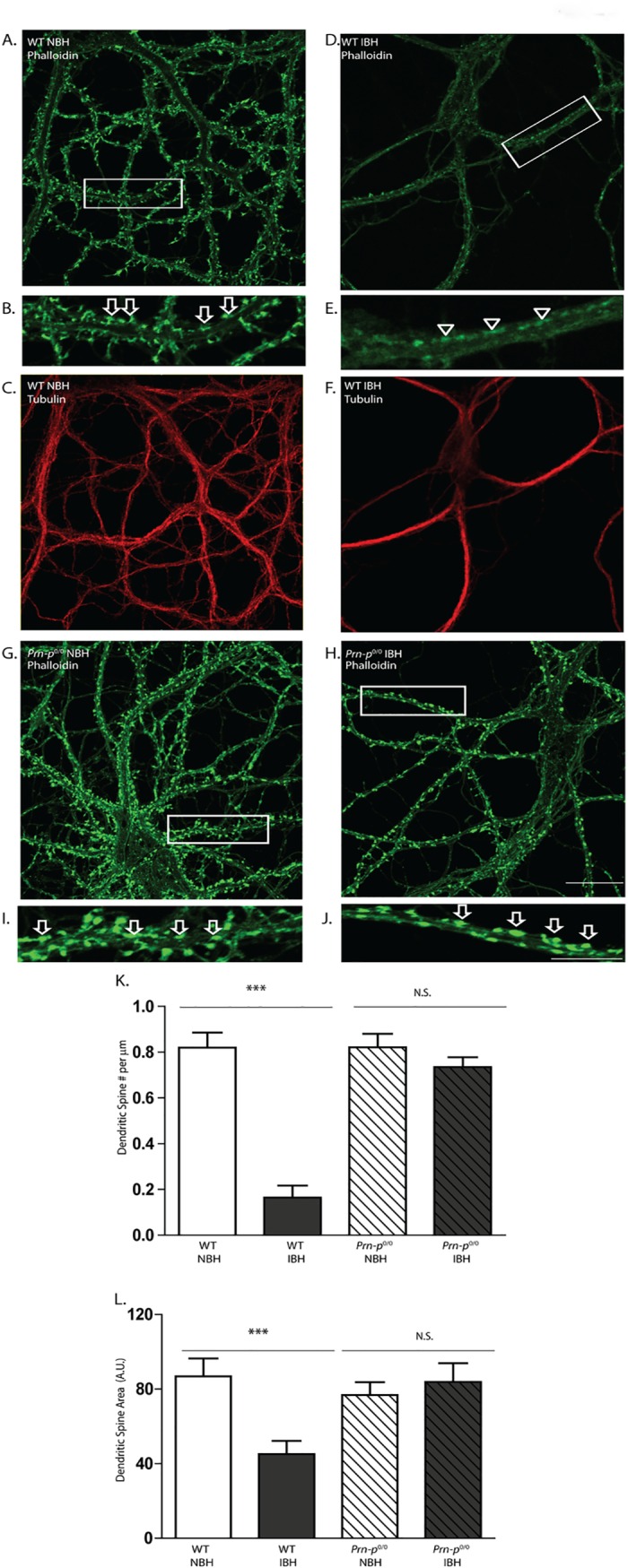
Scrapie-infected brain homogenate causes PrP^C^-dependent loss of dendritic spines. Primary hippocampal neurons from wild-type (WT) mice (**A-F**) or PrP knockout (*Prn-p* ^0/0^) mice (**G-J**) were treated for 24 hr with brain homogenate (0.16% [w/v] final concentration) prepared from either normal mice (NBH) (**A-C, G, I**) or from terminally ill, scrapie-infected mice (IBH) (**D-F, H, J**). Neurons were then fixed and stained with Alexa 488-phalloidin (green) (**A, B, D, E, G-J**) to visualize F-actin, which is enriched in dendritic spines; and with anti-tubulin (red) (**C, F**) to visualize overall dendritic morphology. The boxed regions in panels A, D, G, and H are shown at higher magnification in panels B, E, I, and J, respectively. Arrows in panels B, I, and J point to dendritic spines, and arrowheads in panel E indicate the positions of spines that have retracted. Scale bar in panel H = 20 μm (applicable to panels A, C, D, F, G and H); scale bar in panel J = 10 μm (applicable to panels B, E, and I). Pooled measurements of spine number (**K**) and area (**L**) were collected from 16 neurons from 4 independent experiments for each genotype and each treatment. Spine number is expressed per μm length of dendrite, and spine area as the average area of an individual spine in arbitrary units (A.U.). ***p<0.001 by Student’s t-test; N.S., not significantly different. The decrease in spine area (**L)** reflects the fact that spines gradually shrink prior to completely disappearing; the magnitude of this effect is typically less than the reduction in the number of spines (**K)**.

We found that treatment with IBH significantly altered the morphology of dendritic spines (Fig 1D and 1E). There was dramatic retraction of spines, reducing their number per unit length of dendrite, as well as the area of each dendritic spine (Fig 1K and 1L). Spine retraction was accompanied by collapse of the actin cytoskeleton, resulting in residual patches of Alexa 488-phalloidin staining along the dendrite at the former sites of spines (Fig 1D and 1E). Importantly, the microtubule structure of these neurons remained intact in both dendrites and axons, as indicated by tubulin staining (Fig 1F), demonstrating that IBH was causing an early and selective loss of spines prior to major alterations of neuronal morphology or cell death. Normal brain homogenate (NBH) from age-matched, uninoculated mice had no detectable effect on dendritic spines (comparable with untreated cultures: 0.78 ± 0.03 spines per μm; 81±9.4 A.U. average spine area), suggesting that the toxicity observed was specific to scrapie-infected homogenate (Fig 1A, 1B, 1C, 1K and 1L). To test whether endogenous PrP^C^ is needed for the toxic effect of IBH on dendritic spines, we treated neurons from PrP knock-out (*Prn-p* ^0/0^) mice with IBH. In contrast to wild-type neurons, *Prn-p* ^0/0^ neurons showed no significant change in spine number or area after treatment with IBH (Fig 1G–1L).

As an alternative method to visualize dendritic spines, we infected neurons with a lentivirus encoding GFP, which fills the neuronal cytoplasm, including the inside of spines. We found that IBH induced the shrinkage and disappearance of the GFP-labeled dendritic spines, correlating with changes in phalloidin staining (S1 Fig). This procedure made it clear that dendritic spines were actually retracting and disappearing in response to IBH, and that patches of actin were present at the sites of the collapsed spines.

We conclude that scrapie-infected brain homogenate causes a rapid, PrP^C^-dependent retraction of dendritic spines with little effect on overall dendritic morphology.

### PrP^Sc^ is responsible for dendritic spine retraction

We performed several kinds of experiments to demonstrate that PrP^Sc^ is the component of IBH that causes dendritic spine loss, and that other toxic molecules (e.g. cytokines generated as a result of infection) are not responsible. First, we took advantage of the fact that the N-terminal domain of PrP^C^ is known to bind specifically to PrP^Sc^, even in a complex mixture of proteins[21–24]. We mixed recombinant PrP 23–110 with IBH prior to treatment of hippocampal neurons, with the expectation that the recombinant protein would bind to PrP^Sc^ in the brain homogenate and render it incapable of interacting with PrP^C^ on the neuronal cell surface to produce toxic effects. Consistent with this prediction, we found that addition of PrP 23–110 neutralized the ability of IBH to reduce dendritic spine number and area (S2 Fig).

**Fig 2:**
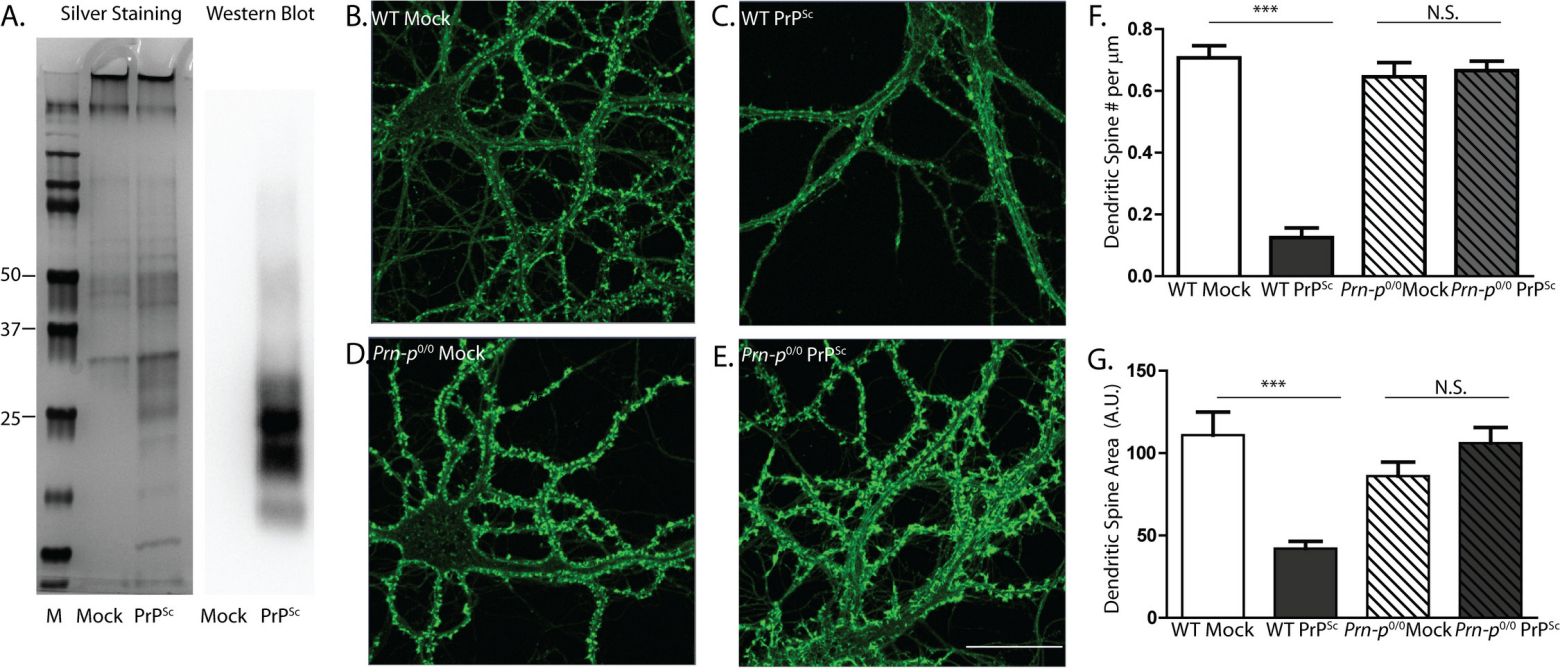
Purified PrP^Sc^, prepared without proteases, causes PrP^C^-dependent spine loss. (**A**) Silver stain and Western blot analysis (using anti-PrP antibody D18) of PrP^Sc^ purified from scrapie-infected brains without proteases, and mock-purified material from uninfected brains. Lane M, molecular size markers in kDa. Hippocampal neurons from wild-type (WT) mice (**B, C**) and PrP knockout (*Prn-p* ^0/0^) mice (**D, E**) were treated for 24 hr with 4.4 μg/ml of purified PrP^Sc^ (**C, E**), or with an equivalent amount of material mock-purified from uninfected brains (**B, D**). Neurons were then fixed and stained with Alexa 488-phalloidin. Scale bar in panel E = 20 μm (applicable to panels B-D). Pooled measurements of spine number (**F**) and area (**G**) were collected from 22–25 cells from 4 independent experiments. ***p<0.001 by Student’s t-test; N.S., not significantly different.

We also tested the effect of two different purified preparations of PrP^Sc^. First, we purified PrP^Sc^ from RML-infected mouse brains in the absence of protease treatment. These preparations, which we estimate to be >50% pure (Fig 2A), caused significant loss of dendritic spines and reduction in area of the remaining spines (Fig 2C, 2F and 2G). Using quantitative dot blotting, we estimated that the final concentration of purified PrP^Sc^ in the medium used to treat the neurons was 4.4 ± 1.1 μg/ml, comparable to the final concentration of PrP^Sc^ in experiments with crude brain homogenate (7.5 ± 2.4 μg/ml). These effects were seen on wild-type neurons, but not on *Prn-p* ^0/0^ neurons (Fig 2E–2G). Samples prepared from uninoculated brains by the same series of steps had no effect (Fig 2B, 2D, 2F and 2G).

**Fig 3:**
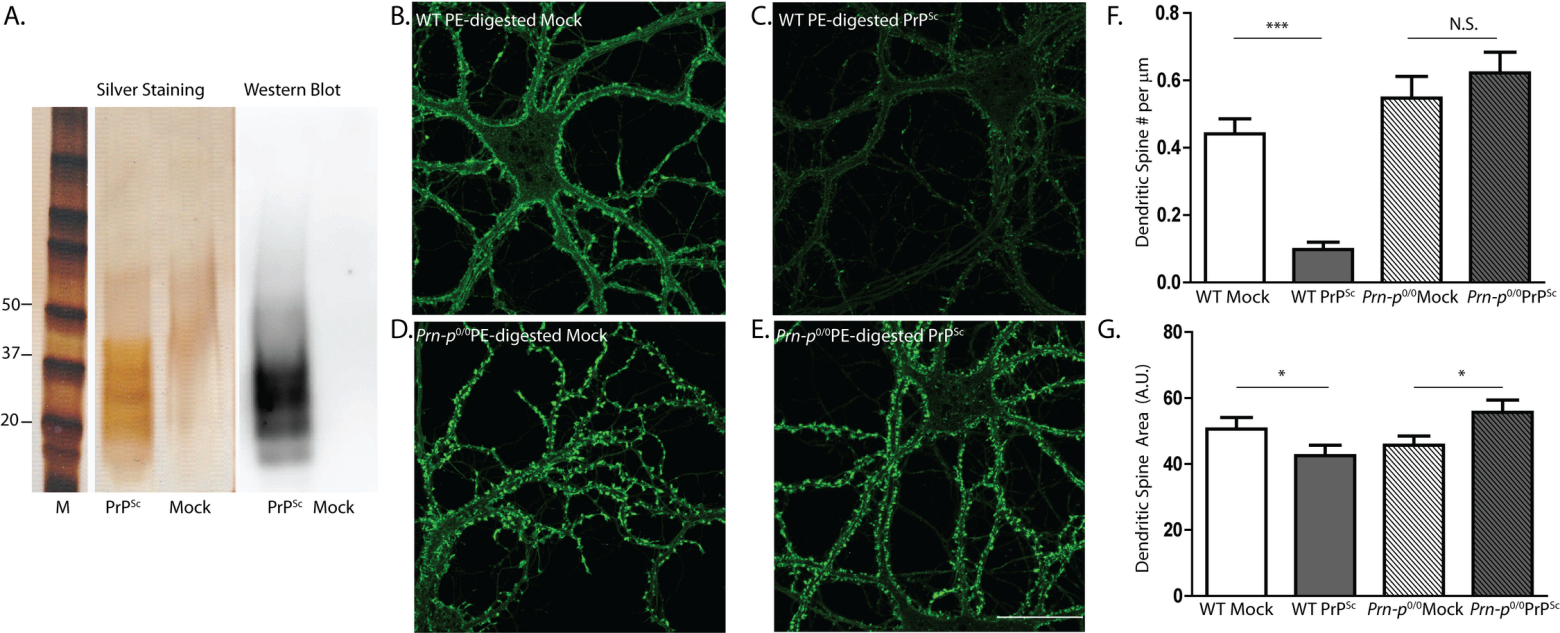
Purified PrP^Sc^, prepared using pronase E, causes PrP^C^-dependent spine loss. (**A**) Silver stain and Western blot analysis (using anti-PrP antibody IPC1) of PrP^Sc^ purified from scrapie-infected brains using pronase E, and mock-purified material from uninfected brains. Lane M, molecular size markers in kDa. Hippocampal neurons from wild-type (WT) mice (**B, C**) and PrP knockout (*Prn-p* ^0/0^) mice (**D, E**) were treated for 24 hr with 4.4 μg/ml of purified PrP^Sc^ (**C, E**), or with an equivalent amount of material mock-purified from uninfected brains (**B, D**). Neurons were then fixed and stained with Alexa 488-phalloidin. Scale bar in panel E = 20 μm (applicable to panels B-D). Pooled measurements of spine number (**F**) and area (**G**) were collected from 16–18 cells from 3 independent experiments. ***p<0.001 or *p<0.05 by Student’s t-test; N.S., not significantly different.

We also purified PrP^Sc^ using a procedure that involves treatment with pronase E followed by precipitation with sodium phosphotungstic acid (NaPTA) in presence of detergent, which efficiently removes PrP^C^, but leaves PrP^Sc^ intact. This procedure results in preparations of higher purity (>90%; Fig 3A) because of proteolysis of contaminating proteins. Pronase E digestion preserves protease-sensitive forms of PrP^Sc^, which are typically digested by the proteinase K included in many PrP^Sc^ purification methods. We treated neurons with a final concentration of pronase E-purified PrP^Sc^ (~4.4 μg/ml) that was equivalent to that of the PrP^Sc^ purified without protease. Pronase E-purified PrP^Sc^ caused significant loss of dendritic spines and reduction in the area of the remaining spines, effects that were seen on wild-type neurons, but not on *Prn-p* ^0/0^ neurons (Fig 3C and 3E–3G). Samples prepared from uninfected brain by the same series of steps had no effect (Fig 3B, 3D, 3F and 3G).

Taken together, these results argue strongly that PrP^Sc^ is the component in our brain-derived preparations that is responsible for the toxic effects on hippocampal dendritic spines, and that these effects are dependent on expression of endogenous PrP^C^ by the target neurons.

### Proteinase K-treated PrP^Sc^ retains synaptotoxicity

**Fig 4:**
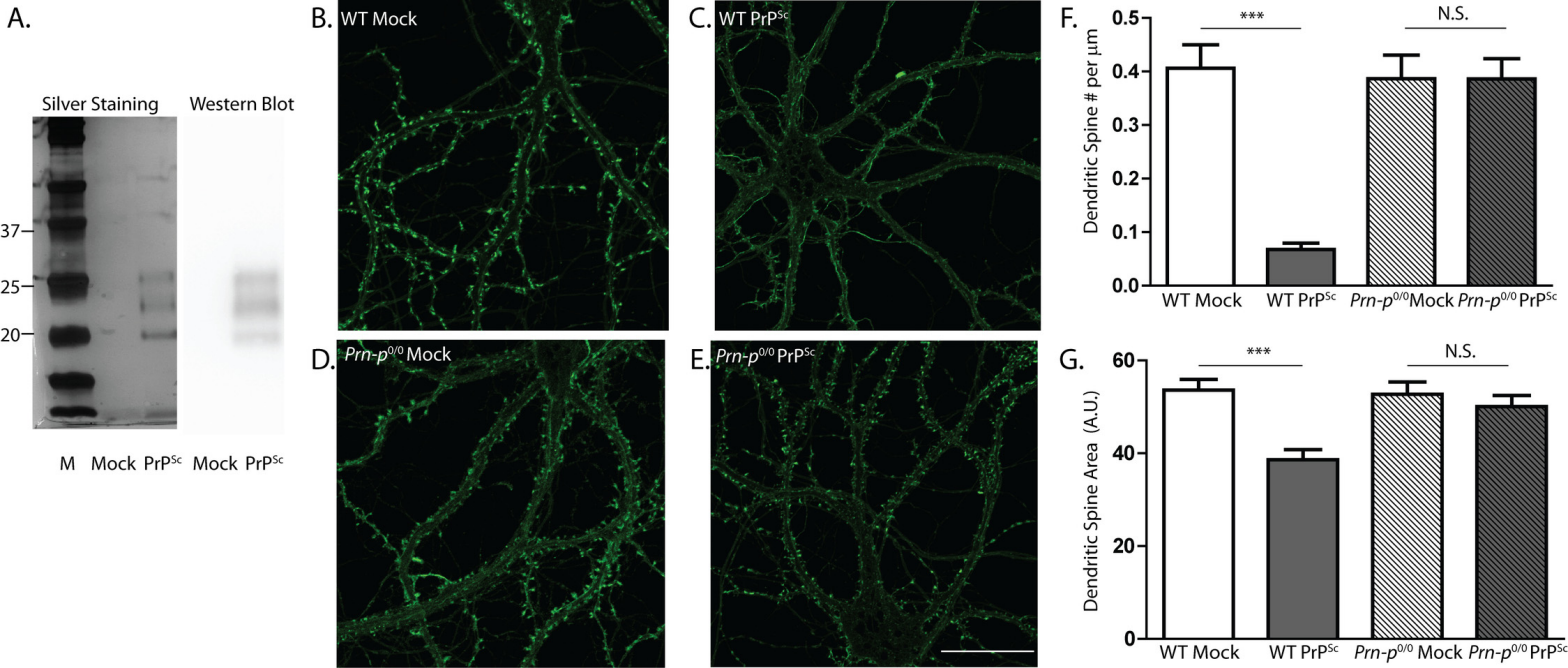
PK-digested PrP^Sc^ causes dendritic spine loss. (**A**) Silver stain and Western blot (using anti-PrP antibody D18) of a PrP^Sc^ sample and a mock-purified control sample, after digestion with PK. Lane M, molecular size markers in kDa. Hippocampal neurons from wild-type (WT) mice (**B, C**) and PrP knockout (*Prn-p* ^0/0^) mice (**D, E**) were treated for 24 hr with 4.4 μg/ml of purified, PK-treated PrP^Sc^ (**C, E**), or with an equivalent amount of mock-purified sample (**B, D**). Neurons were then fixed and stained with Alexa 488-phalloidin. Scale bar in panel E = 20 μm (applicable to panels B-D). Pooled measurements of spine number (**F**) and area (**G**) were collected from 20–24 cells from 3 independent experiments. ***p<0.001 by Student’s t-test; N.S., not significantly different.

PrP^Sc^ purified from brain typically consists of both PK-resistant and PK-sensitive species, which may have different toxic properties. We therefore tested the effect of PK-treated PrP^Sc^ on dendritic spine integrity. Based on quantitative Western blotting, PK treatment resulted in digestion of ~90% of the PrP^Sc^, which represents PK-sensitive PrP^Sc^. This proportion of PK-sensitive PrP^Sc^ is similar to that reported in other studies[25]. As shown in Fig 4A, treatment with PK resulted in a highly purified preparation of PrP^Sc^ (>95% purity, based on silver staining), due to the digestion of non-specific proteins. Treatment with PK also resulted in a downward shift in size due to removal of the N-terminal ~65 amino acids. When we tested the toxicity of PK-digested PrP^Sc^ at a concentration of 4.4 μg/ml, equivalent to the concentration of non-PK-treated material used in Fig 2, we observed roughly comparable effects: approximately 85% reduction in spine density and 30% reduction in spine area, with no significant effect on *Prn-p* ^0/0^ neurons, and no effect of mock-purified material from uninfected brains (Fig 4B–4G). These results indicate that residues 23 through ~90 of PrP^Sc^ are not essential for synaptotoxicity. Moreover, since similar toxic effects were seen with equivalent concentrations of undigested and PK-digested PrP^Sc^, our data suggest that both PK-sensitive and PK-resistant forms of PrP^Sc^ may contribute to dendritic spine loss.

### The toxic effect of PrP^Sc^ is dose-dependent

The concentration of purified PrP^Sc^ used in the experiments shown in Figs 2–4 was 4.4 μg/ml, similar to the estimated concentration of PrP^Sc^ in the crude IBH used in Fig 1. To determine the minimum PrP^Sc^ concentration required to observe a toxic effect on dendritic spines, we tested the dose dependence using hippocampal neurons from wild-type mice, as well as Tga20 mice which express ~10X the endogenous level of wild-type PrP^C^ (S3 Fig). At the highest concentration tested (4.4 μg/ml), purified PrP^Sc^ reduced spine density by ~85% and spine area by ~30%. Smaller, but statistically significant effects (50% and 25% reduction in spine density and area, respectively) were seen with 2.2 μg/ml PrP^Sc^. At 1.1 μg/ml PrP^Sc^, there were trends for a reduction in spine density and area, but the differences were not statistically significant. At each concentration of PrP^Sc^, there was no significant difference between WT and Tga20 neurons. As a control, a mock-purified preparation from uninfected brain, equivalent to the highest PrP^Sc^ concentration, had no effect on spine density or area. Taken together, these results indicate that synaptotoxic effects of PrP^Sc^ can be detected at concentrations as low as 2.2 μg/ml, and that boosting the expression of PrP^C^ beyond the endogenous level does not increase the degree of toxicity.

### The N-terminal domain of PrP^C^ is essential for PrP^Sc^-induced dendritic spine loss

**Fig 5:**
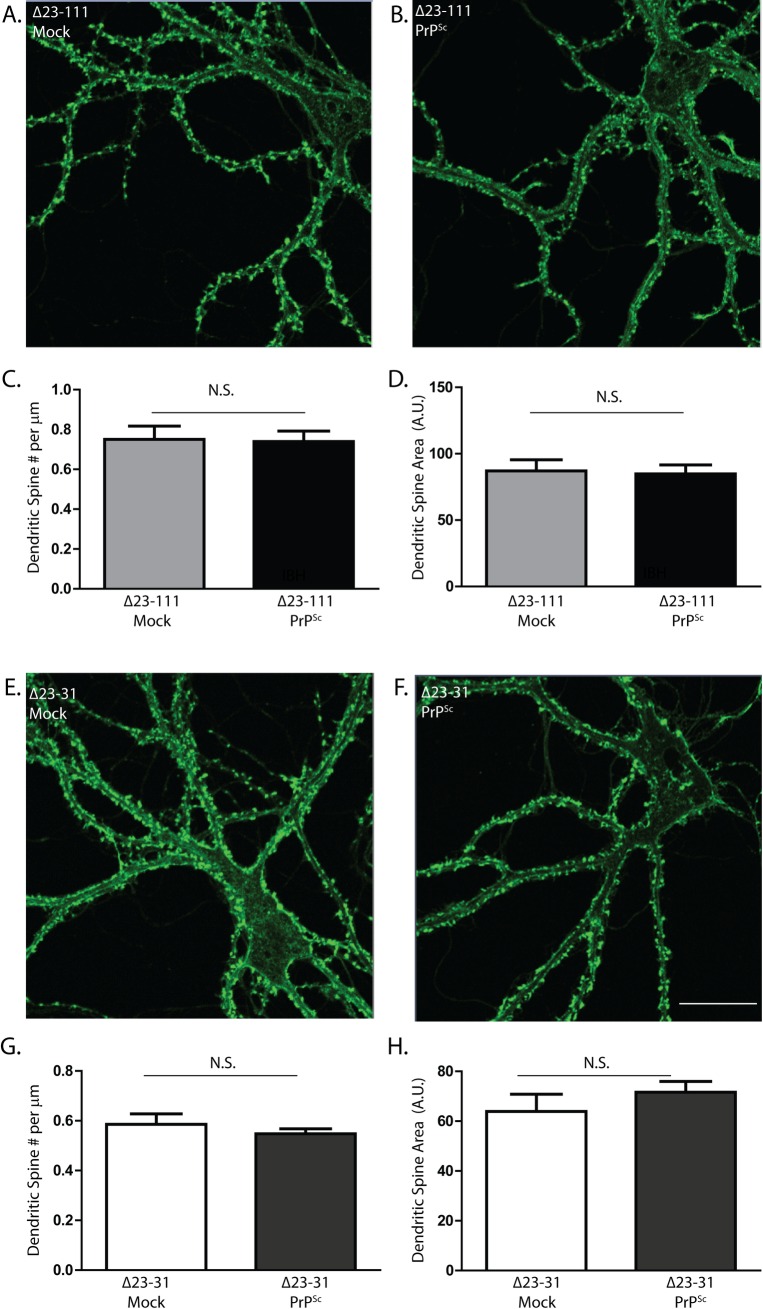
The N-terminal domain of PrP^C^ is essential for PrP^Sc^-induced dendritic spine loss. Hippocampal neurons from Tg(Δ23–111) mice (**A-D**) and Tg(Δ23–31) mice (**E-H**) (both on the *Prn-p* ^0/0^ background) were treated for 24 hr with 4.4 μg/ml of PrP^Sc^ purified without proteases (**B, F**), or with an equivalent amount of mock-purified material from uninfected brains (**A, E**). Neurons were then fixed and stained with Alexa 488-phalloidin. Scale bar in panel F = 20 μm (applicable to panels A, B, E). Pooled measurements of spine number (**C, G**) and area (**D, H**) were collected from 20–24 cells from 4 independent experiments. N.S., not significantly different by Student’s t-test.

Our previous results indicated that PrP^C^ expression by target neurons is essential for dendritic spine loss induced by PrP^Sc^. We went on to analyze which regions of PrP^C^ are critical for this effect. We prepared hippocampal neurons from two lines of transgenic mice that we have previously constructed: Tg(Δ23–111) and Tg(Δ23–31), which express PrP^C^ harboring deletions of residues 23–111 and 23–31, respectively. These mice do not express endogenous PrP (i.e. they have a *Prn-p* ^0/0^ background), and the expression levels of the Δ23–111 and Δ23–31 proteins are, respectively, 7X[26] and 1x[27] the endogenous PrP levels found in WT mice. We found that neurons from both lines of transgenic mice were completely resistant to the toxic effects of purified PrP^Sc^, with dendritic spine density and area similar to those of neurons treated with mock-purified material from uninfected brains (Fig 5). These results indicate that a small, polybasic region of PrP^C^ (residues 23–31) expressed on target neurons is essential for spine loss induced by exogenously applied PrP^Sc^.

## Discussion

In this study, we have established an experimental system to detect the synaptotoxic effects of PrP^Sc^ based on its ability to cause retraction and loss of dendritic spines on cultured hippocampal neurons. We have shown that crude brain homogenates from scrapie-infected mice, as well as three kinds of purified preparations of PrP^Sc^ cause dendritic spine retraction, while similar preparations from normal, uninfected brains have no effect. These results argue strongly that PrP^Sc^ itself, rather than other toxic molecules present in infected brain are responsible for the dendritic spine loss in our system. We suggest that our system is registering relatively early events in the pathogenic cascade triggered by PrP^Sc^. Retraction of spines is detectable within 24 hours of PrP^Sc^ exposure, and occurs prior to other changes in overall dendritic morphology or neuronal cell death. Importantly, the ability of PrP^Sc^ to cause dendritic spine loss is entirely dependent on expression of PrP^C^ by target neurons, and on a small, polybasic region at the N-terminus of the PrP^C^ molecule (residues 23–31; KKRPKPGGW).

Our system reproduces the earliest changes in dendritic spine morphology that have been observed in the brains of living, scrapie-infected mice by two-photon imaging[12], as well as in organotypic slice cultures and fixed, brain sections[6–11]. We therefore believe that our experimental approach reveals mechanisms that are directly relevant to pathological processes that occur *in vivo* during prion diseases. Spine retraction in our system is accompanied by a major collapse of the actin cytoskeleton of the spine, consistent with the dynamic role of actin filaments in maintenance of spine morphology[28]. As shown in this study, our system has already provided several new insights into prion neurotoxicity, and in the future it promises to further illuminate the underlying cellular and molecular mechanisms, thus leading to identification of potential, new therapeutic targets.

PrP^Sc^-induced loss of dendritic spines requires the expression of PrP^C^ on the target neurons, most likely because cell-surface PrP^C^ acts as receptor to bind exogenously added PrP^Sc^. This suggestion is consistent with the observation that neuronal expression of membrane-anchored PrP^C^ is necessary for prion-induced neurodegeneration *in vivo*[29–32]. One possible scenario is that dendritic loss results directly from binding of PrP^Sc^ to PrP^C^, with PrP^C^ itself acting as a toxicity-transducing receptor. An alternative but not mutually exclusive possibility is that cell surface PrP^C^ is first converted to PrP^Sc^ (or some other misfolded form) which then elicits a toxic signal. The latter possibility would be consistent with a recent report[33] that cell surface PrP^C^ is converted to PrP^Sc^ within minutes of contact with exogenously applied PrP^Sc^. Interestingly, we found that Tga20 neurons, which express 10X the endogenous level of PrP^C^, are no more susceptible than WT neurons to the spine-retracting effects of PrP^Sc^. This suggests that the expression level of PrP^C^ is not the rate-limiting step in this initial pathological process.

Our results highlight a critical role for the N-terminal domain of PrP^C^, particularly polybasic residues 23–31, in transducing the synaptotoxic effects of PrP^Sc^. Remarkably, neurons expressing exclusively PrP^C^ molecules missing residues 23–31 or 23–111 are completely resistant to dendritic spine loss induced by PrP^Sc^. This result could be attributable to the previously documented role of residues 23–31 in PrP^C^ binding to PrP^Sc^ [22–24]. Alternatively, the N-terminal domain may play a direct role in the ability of PrP^C^ or PrP^Sc^ to elicit downstream neurotoxic signals. This hypothesis would be consistent with the requirement for the 23–31 region in the neurodegenerative phenotype of transgenic mice expressing certain PrP deletion mutants, as well as for the spontaneous ion channel activity associated with these mutants[34,35]. The N-terminal domain has also been shown to be essential for the ability of certain anti-PrP antibodies to elicit neuronal cell death in brain slices [36].

The results presented here shed light on the nature of the toxic species responsible for prion neurotoxicity. PrP^Sc^ purified from brain is known to be heterogeneous in terms of aggregation state, protease resistance, and possibly protein conformation [25,37–40]. Moreover, there is evidence that infectivity (the ability to self-propagate) and neurotoxicity (the ability to produce neuropathology) may be distinct properties attributable to different molecular forms of misfolded PrP[41–44]. Although historically PK resistance has been used to define PrP^Sc^ in biochemical analyses, it has been estimated that a large fraction of the PrP^Sc^ present in the brain after prion infection is actually sensitive to PK digestion [25,38–40]. There is debate about the relative infectivity of the PK-resistant and PK-sensitive forms, and it has been suggested that the latter species may represent small aggregates that are particularly neurotoxic without being infectious [41,43]. We have demonstrated here that both protease-sensitive and protease-resistant forms of PrP^Sc^ have synaptotoxic activity in our assay, and that the N-terminal domain of PrP^Sc^ that is removed by PK treatment is not essential for this activity. These results suggest that multiple molecular forms of PrP^Sc^ differing in aggregation state and quaternary structure may possess neurotoxic activity.

Recently, Aguzzi and colleagues have described an organotypic cerebellar slice system in which scrapie infection results in neuronal death, and they have used this system to study some of the cellular mechanisms underlying prion neurotoxicity[7,45,46]. This system requires chronic infection of the slices with scrapie, which takes several weeks, and is therefore likely to be registering pathological changes secondary to generalized neuronal loss, in contrast to the earlier, more specific dendritic spine alterations observed in our assay.

Our neuronal culture system will make it possible to address several outstanding issues in prion biology. These include identification of the downstream signaling mechanisms that link PrP^Sc^ binding by PrP^C^ on the cell surface to neurotoxic sequelae such as dendritic spine retraction, characterization of electrophysiological alterations in synaptic function caused by PrP^Sc^, and analysis of the molecular determinants of neurotoxic vs. infectious forms of PrP. This system may also allow identification of new therapeutic targets, and the testing of compounds that act directly on neurotoxic signaling pathways rather than on the formation of PrP^Sc^.

Finally, this system will allow direct comparisons between pathogenic mechanisms involved in prion diseases and other neurodegenerative disorders. Dendritic spine loss is a common theme in many neurodegenerative conditions, including Alzheimer’s, Huntington’s, and Parkinson’s diseases, and has been suggested to contribute to clinical symptoms in patients[47]. Of note, changes in dendritic morphology in cultured hippocampal neurons have been widely used as an experimental readout of the synaptotoxicity of the Alzheimer’s Aβ peptide[48]. Since Aβ oligomers have been shown to bind to PrP^C^, and to induce dendritic spine retraction that appears analogous to that produced by PrP^Sc^ [49–53], it will be of interest to investigate whether the two neurotoxic aggregates act by similar mechanisms.

## Methods

### Primary neuronal cultures

*Prn-p* ^0/0^ mice[54] and Tga20 mice[55] on a C57BL6 background were obtained from the European Mouse Mutant Archive (EMMA; Rome, Italy), and were maintained in a homozygous state by interbreeding. Tg(Δ23–111) mice[26] (also referred to as Tg(C1)) and Tg(Δ23–31) mice[27] were constructed as described, and maintained on a *Prn-p* ^0/0^ background with the transgene array in a hemizygous state. Timed-pregnant C57BL/6 mice (referred to as wild-type, WT) were purchased from the Jackson Laboratory (Bar Harbor, ME). Mice were genotyped by PCR analysis of tail DNA prepared using the Puregene DNA Isolation kit (Gentra Systems) using primers as described previously[26,27]. All procedures involving animals were conducted according to the United States Department of Agriculture Animal Welfare Act and the National Institutes of Health Policy on Humane Care and Use of Laboratory Animals.

Hippocampal neurons were cultured from P0 pups as described[20]. Neurons were seeded at 75 cells/mm^2^ on poly-L-lysine-treated coverslips, and after several hours the coverslips were inverted onto an astrocyte feeder layer and maintained in NB/B27 medium until used. The astrocyte feeder layer was generated using murine neural stem cells, as described[56]. Neurons were kept in culture for 18–21 days prior to PrP^Sc^ treatment.

### Ethics statement

All procedures involving animals were conducted according to the United States Department of Agriculture Animal Welfare Act and the National Institutes of Health Policy on Humane Care and Use of Laboratory Animals. Ethical approval (AN-14997) was obtained from Boston University medical center institutional animal care and use committee.

### Immunostaining and dendritic spine quantitation

Neurons were treated with PrP^Sc^-containing or control preparations for 24 hr, followed by fixation in 4% paraformaldehyde and staining with either Alexa 488-phalloidin or rhodamine-phalloidin (ThermoFischer Scientific, Waltham, MA) to visualize dendritic spines, and anti-tubulin antibodies (Sigma-Aldrich, St. Louis, MO) to visualize axons and dendrites. Images were acquired using a Zeiss 700 confocal microscope with a 63x objective (N.A. = 1.4). The number and area of dendritic spines were determined using ImageJ software. Briefly, 2–3 dendritic segments with a clear background were chosen from each image, and the images adjusted using a threshold that had been optimized to include the outline of the spines but not non-specific signals[57]. Spine area and number were determined. The number of spines was normalized to the measured length of the dendritic segment to give the number of spines/μm, and the area was normalized to the number of spines to give the average area of each spine in arbitrary units (A.U.). For each experiment, 15–24 neurons from 3–4 individual experiments were imaged and quantitated.

### Lentiviral infection

A GFP-encoding lentivirus (created using the vector CSCW-Fluc-IRES-GFP) was obtained from the Massachusetts General Hospital Vector Core (https://vectorcore.mgh.harvard.edu). Virus (10^5^ IU/ml final concentration) was added to hippocampal neurons after 12 days in culture. Neurons were then cultured for an additional 6 days, to maximize GFP expression, before PrP^Sc^ treatment.

### Scrapie inoculation of mice

C57BL6 or FVB mice (as indicated below) were inoculated intracerebrally with 30 μl of a 1% brain homogenate from the brain of a corresponding mouse that was terminally ill with RML scrapie. The inoculated mice were monitored until the appearance of clinical signs (~170 days post inoculation), at which time the animals were euthanized, their brains collected, and stored at -80°C until use.

### Preparation of brain homogenates for treatment of cultured neurons

Ten percent (w/v) brain homogenates in PBS were prepared from RML infected or non-infected C57BL6 mice using 1 mm glass beads (D1031-10, Benchmark Scientific, Edison, NJ) and a Beadbug microtube homogenizer (Benchmark Scientific). Brain homogenates were passed once through a 0.5 cc insulin syringe with a 28 gauge needle (Becton Dickinson, cat. no. 329461), and aliquoted for storage at -80°C.

### Purification of full-length PrP^Sc^

Two different procedures were used to purify full-length PrP^Sc^ from the brains of terminally ill mice infected with the RML strain of scrapie: one that did not use proteases; and another that employed pronase E, which preserves full-length PrP^Sc^. Mock purifications were also carried out from age-match, uninfected brains. The purified samples were evaluated by SDS-PAGE followed by silver staining and Western blotting.

#### Protease-free method

This procedure was carried out as previously described[43]. In a typical preparation, 18 RML-infected C57BL6 brains were homogenized in 3 ml of 10% sarkosyl in TEND (10 mM Tris-HCl [pH 8], 1 mM EDTA, 130 mM NaCl, and 1 mM dithiothreitol) containing Complete Protease Inhibitor Cocktail (Roche Diagnostics, cat. no. 11836153001) using a glass bead homogenizer as described above. Brain homogenates were incubated on ice for 1 hr and centrifuged at 22,000 x g for 30 min at 4°C. The supernatant was kept on ice, while the pellet was resuspended in 1 ml of 10% sarkosyl in TEND, incubated for 1 hr on ice, and then centrifuged at 22,000 x g for 30 min at 4°C. The pellet was discarded while the supernatants were pooled and centrifuged at 150,000 x g for 2.5 h at 4°C. The new supernatants were discarded, while the pellets were rinsed with 50 ml of 100 mM NaCl, 1% sulfobetaine (SB) 3–14 in TEND plus protease inhibitors, and then pooled by resuspending them in 1 ml of the wash buffer, and centrifuging at 180,000 x g for 2 hr at 20°C. The supernatant was discarded, and the pellet was rinsed with 50 ml of TMS (10 mM Tris-HCl [pH 7.0], 5 mM MgCl_2_, and 100 mM NaCl) plus protease inhibitors, resuspended in 600 μl of the same buffer containing 100 mg/ml RNase A and incubated for 2 hr at 37°C. The sample was then incubated with 5 mM CaCl_2_, 20 mg/ml DNase I for 2 hr at 37°C. To stop the enzymatic digestion, EDTA was added to a final concentration of 20 mM, and the sample was mixed with an equal volume of TMS containing 1% SB 3–14. The sample was gently deposited on a 100 μl cushion of 1 M sucrose, 100 mM NaCl, 0.5% SB 3–14, and 10 mM Tris-HCl (pH 7.4), and centrifuged at 180,000 x g for 2 hr at 4°C. The supernatant was discarded and the pellet was rinsed with 50 μl of 0.5% SB 3–14 in PBS, resuspended in 1 ml of the same buffer, subjected to 5 X 5 sec pulses of bath sonication with a Bandelin Sonopuls Ultrasonicator (Amtrex Technologies) at 90% power, and centrifuged at 180,000 x g for 15 min at 4°C. The final supernatant was discarded and the final pellet was resuspended in 900 μl of PBS (50μl for each starting brain) and sonicated 5 times for 5 sec. Aliquots were stored at -80°C.

#### Pronase E method

This protocol is based on the precipitation of PrP^Sc^ with sodium phosphotungstate (NaPTA)[58] and limited proteolysis with pronase E[59]. RML-infected FVB mouse brains were homogenized in PBS to generate a 10% (w/v) brain homogenate. After a clarification centrifugation step (500 x g at 4°C for 10 min), the supernatant was incubated with 2% sarkosyl for 1 hr and subsequently digested with 100 μg/ml of pronase E (Protease Type XIV from *Streptomyces griseous*; Sigma Aldrich, cat. no. P5147) for 30 min. The pronase E digestion was stopped with 2 mM PMSF and 10 mM EDTA. Afterwards, the samples were incubated with 0.3% (w/v) NaPTA (pH 7.0) for 1 hr and centrifuged at 16,000 x g and 4°C for 30 min. The pellet was resuspended in 2% sarkosyl and incubated overnight. The next day, the samples were adjusted to 0.3% (w/v) NaPTA and incubated for 1 hr, obtaining the final pellet by centrifugation at 18,000 x g and 4°C for 30 min. The final pellets were resuspended in PBS, with one brain-equivalent being resuspended in 50 μl of PBS. Aliquots were stored at -80°C. All digestions and incubations were performed at 37°C with vigorous agitation.

### PK digestion of purified PrP^Sc^

Purified PrP^Sc^ (prepared by the protease-free method), or the equivalent amount of mock-purified material from uninfected brains, was incubated in a total volume of 250 μl of PBS/2% sarkosyl containing a final concentration of 20 μg/ml of PK (Roche Diagnostics, cat. no. 03115879001) for 1 hr at 37°C. Then 5 μl of 50X Complete Protease Inhibitor Cocktail (Roche Diagnostics, cat. no. 11836153001) was added, followed by 700 μl of PBS. Samples were then centrifuged at 180,000g at 4°C for 1 hr. The supernatant was discarded and the pellet resuspended in 21 μl of PBS. 20 μl was used to treat hippocampal neurons, and the remaining 1 μl was used for analysis by Western blotting and silver staining.

### Western blotting and silver staining

Proteins in SDS sample buffer were heated at 95°C for 5 min, then resolved by SDS-PAGE in 12% pre-cast gels (BioRad, cat. no. 567–1044). For Western blotting, proteins were electrophoretically transferred to PVDF membranes (Millipore, cat. no. IPVH00010). Membranes were blocked for 1 h in 5% (w/v) non-fat dry milk in Tris-buffered saline containing 0.1% Tween 20, followed by incubation with anti-PrP antibody D18 (a human chimeric monoclonal[60]), and then with HRP-conjugated anti-human secondary antibody (Jackson ImmunoResearch, cat. no. 109-035-088). Signals were revealed using HRP substrate (Millipore, cat. no. WBKLS0500), and were visualized using a BioRad XRS image scanner. Silver staining of gels was carried out using a Silver Stain Kit (Pierce/ThermoFisher cat. no. 24612) following the manufacturer’s instructions.

## Supporting Information

S1 Fig Scrapie-infected brain homogenate causes loss of GFP-labeled dendritic spines, consistent with phalloidin staining results. Primary hippocampal neurons from wild-type neurons were transduced with GFP-lentivirus, and were then treated for 24 hr with either normal brain homogenate (NBH) (**A-D**) or scrapie-infected brain homogenate (IBH) (**E-H**). Neurons were then fixed and stained with rhodamine-phalloidin. The same fields were then imaged to reveal either GFP (green) (**A, C, E, G**) or rhodamine-phalloidin (red) (**B, D, F, H**). The boxed regions in panels A, B, E, and F are shown at higher magnification in panels C, D, G, and H, respectively. Arrows in panels C and D point to dendritic spines; arrowheads in panels G and H indicate the positions of spines that have retracted and are marked by residual actin staining (panel H), but are not visible by GFP fluorescence (panel G). Scale bar in panel F = 20 μm (applicable to panels A, B, E); scale bar in panel H = 10 μm (applicable to panels C, D, G).
(TIF)

S2 Fig PrP peptide blocks the ability of scrapie-infected brain homogenate to cause dendritic spine loss. Primary hippocampal neurons from wild-type mice were treated for 24 hr with brain homogenate (0.16% [w/v] final concentration) prepared from either normal mice (NBH) (**A**) or from terminally ill, scrapie-infected mice (IBH) (**B**). Synthetic peptide PrP 23–109 was mixed with NHB or IBH at a final concentration of 167 ng/ml prior to treatment of neurons. Neurons were then fixed and stained with Alexa 488-phalloidin Scale bar in panel B = 20 μm (applicable to panel A). Pooled measurements of spine number (**C**) and area (**D**) were collected from 15–16 neurons from 4 independent experiments for each treatment. N.S., not significantly different by Student’s t-test.
(TIF)

S3 Fig Dendritic toxicity is dependent on PrP^Sc^ concentration, and is not enhanced by over-expression of PrP^C^. Primary hippocampal neurons from wild-type or Tga20 mice were treated for 24 hr with the indicated concentrations of purified PrP^Sc^ (prepared without proteases), or with an equivalent amount of material mock-purified from uninfected brains. Neurons were then fixed and stained with Alexa 488-phalloidin Pooled measurements of spine number (**A**) and area (**B**) were collected from 10–15 neurons from 3 independent experiments for each treatment. ***p<0.001 by Student’s t-test; N.S., not significantly different.
(TIF)
