# Supplementary figures and images for: A Neuronal Culture System to Detect Prion Synaptotoxicity
Source: PLoS Pathog. 2016 May 26;12(5):e1005623. doi: 10.1371/journal.ppat.1005623 (PMC4881977; doi:10.1371/journal.ppat.1005623)

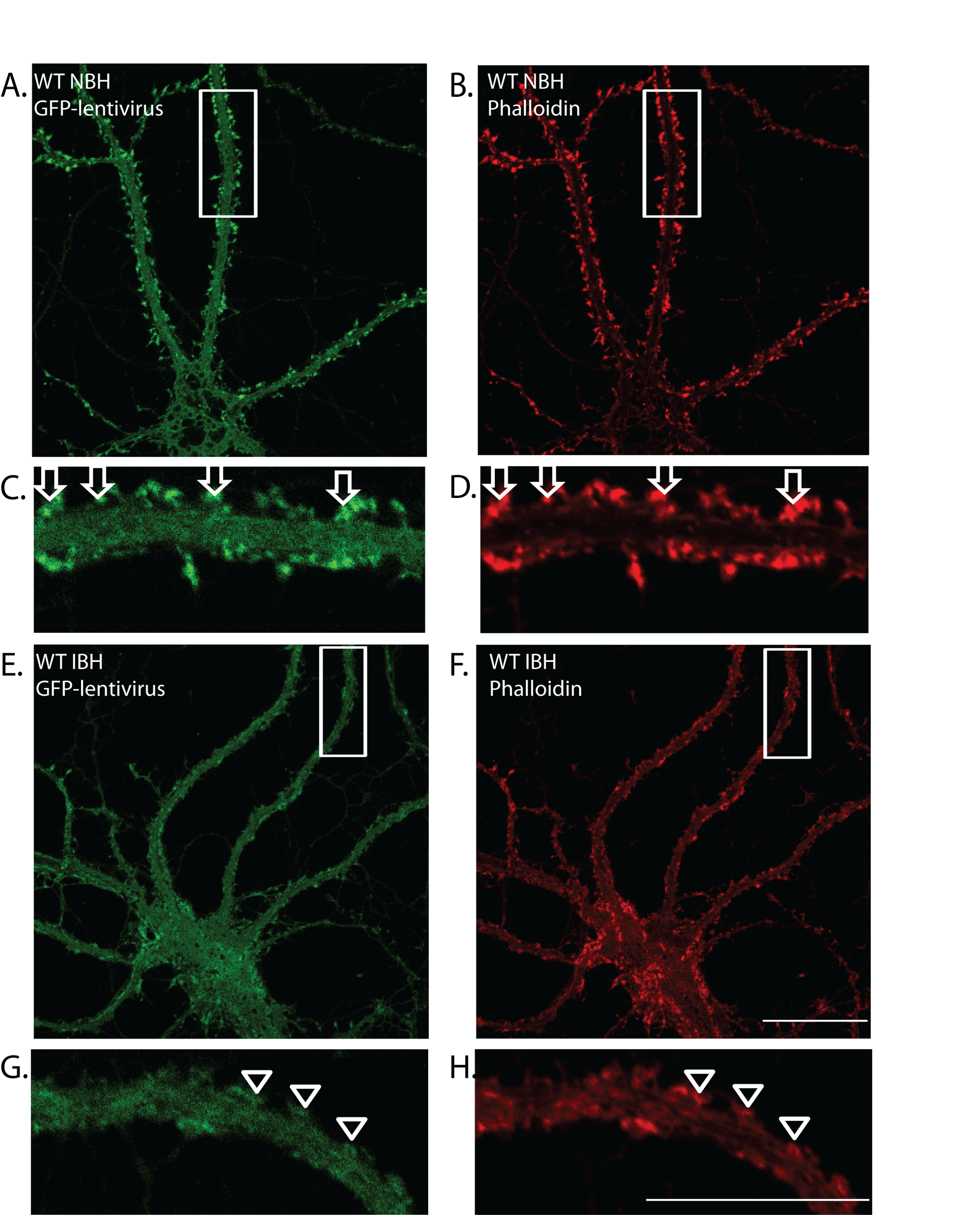

Supplement: S1 Fig — Primary hippocampal neurons from wild-type neurons were transduced with GFP-lentivirus, and were then treated for 24 hr with either normal brain homogenate (NBH) (A-D) or scrapie-infected brain homogenate (IBH) (E-H). Neurons were then fixed and stained with rhodamine-phalloidin. The same fields were then imaged to reveal either GFP (green) (A, C, E, G) or rhodamine-phalloidin (red) (B, D, F, H). The boxed regions in panels A, B, E, and F are shown at higher magnification in panels C, D, G, and H, respectively. Arrows in panels C and D point to dendritic spines; arrowheads in panels G and H indicate the positions of spines that have retracted and are marked by residual actin staining (panel H), but are not visible by GFP fluorescence (panel G). Scale bar in panel F = 20 μm (applicable to panels A, B, E); scale bar in panel H = 10 μm (applicable to panels C, D, G). (TIF) [file ppat.1005623.s001.tif]

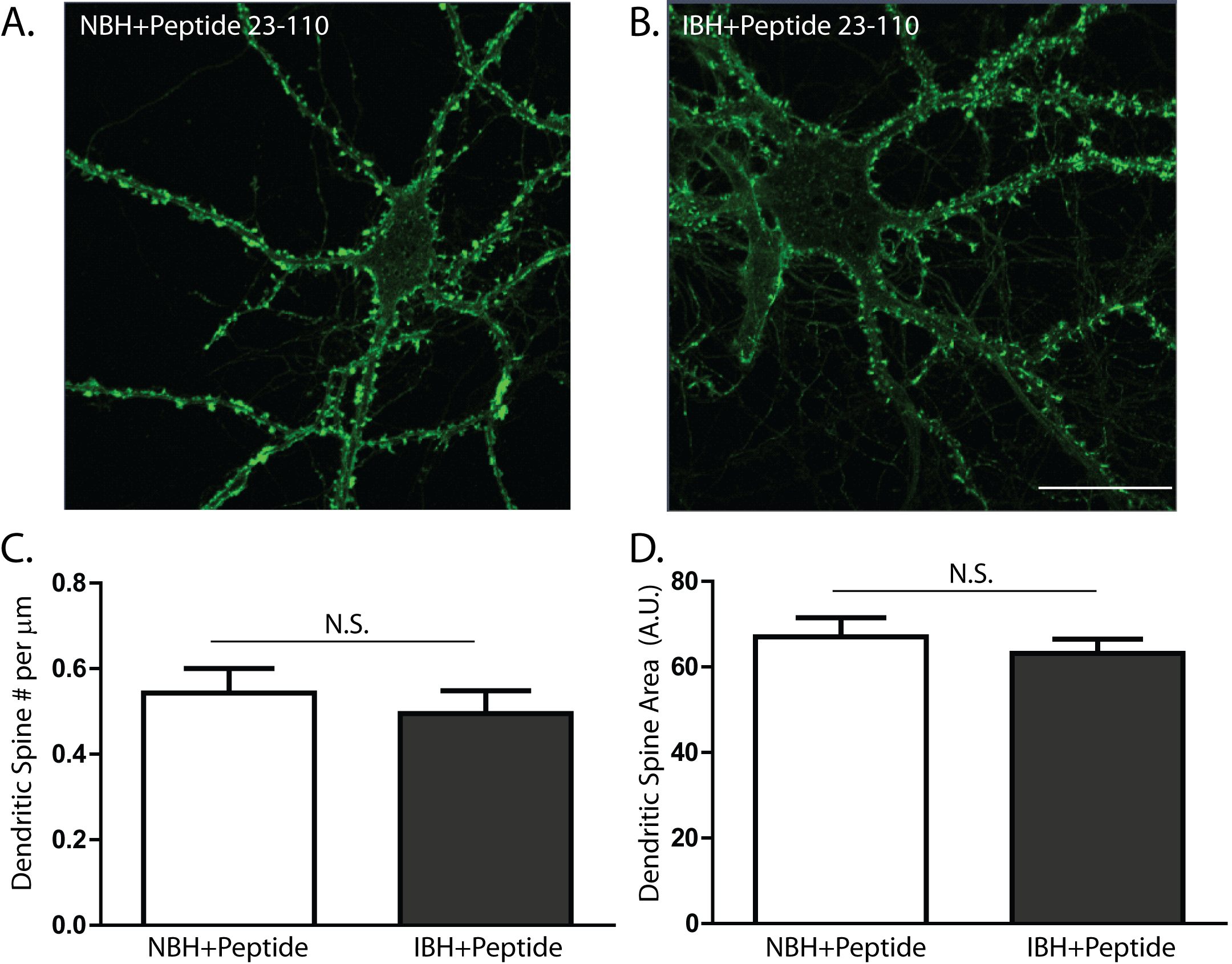

Supplement: S2 Fig — Primary hippocampal neurons from wild-type mice were treated for 24 hr with brain homogenate (0.16% [w/v] final concentration) prepared from either normal mice (NBH) (A) or from terminally ill, scrapie-infected mice (IBH) (B). Synthetic peptide PrP 23–109 was mixed with NHB or IBH at a final concentration of 167 ng/ml prior to treatment of neurons. Neurons were then fixed and stained with Alexa 488-phalloidin Scale bar in panel B = 20 μm (applicable to panel A). Pooled measurements of spine number (C) and area (D) were collected from 15–16 neurons from 4 independent experiments for each treatment. N.S., not significantly different by Student’s t-test. (TIF) [file ppat.1005623.s002.tif]

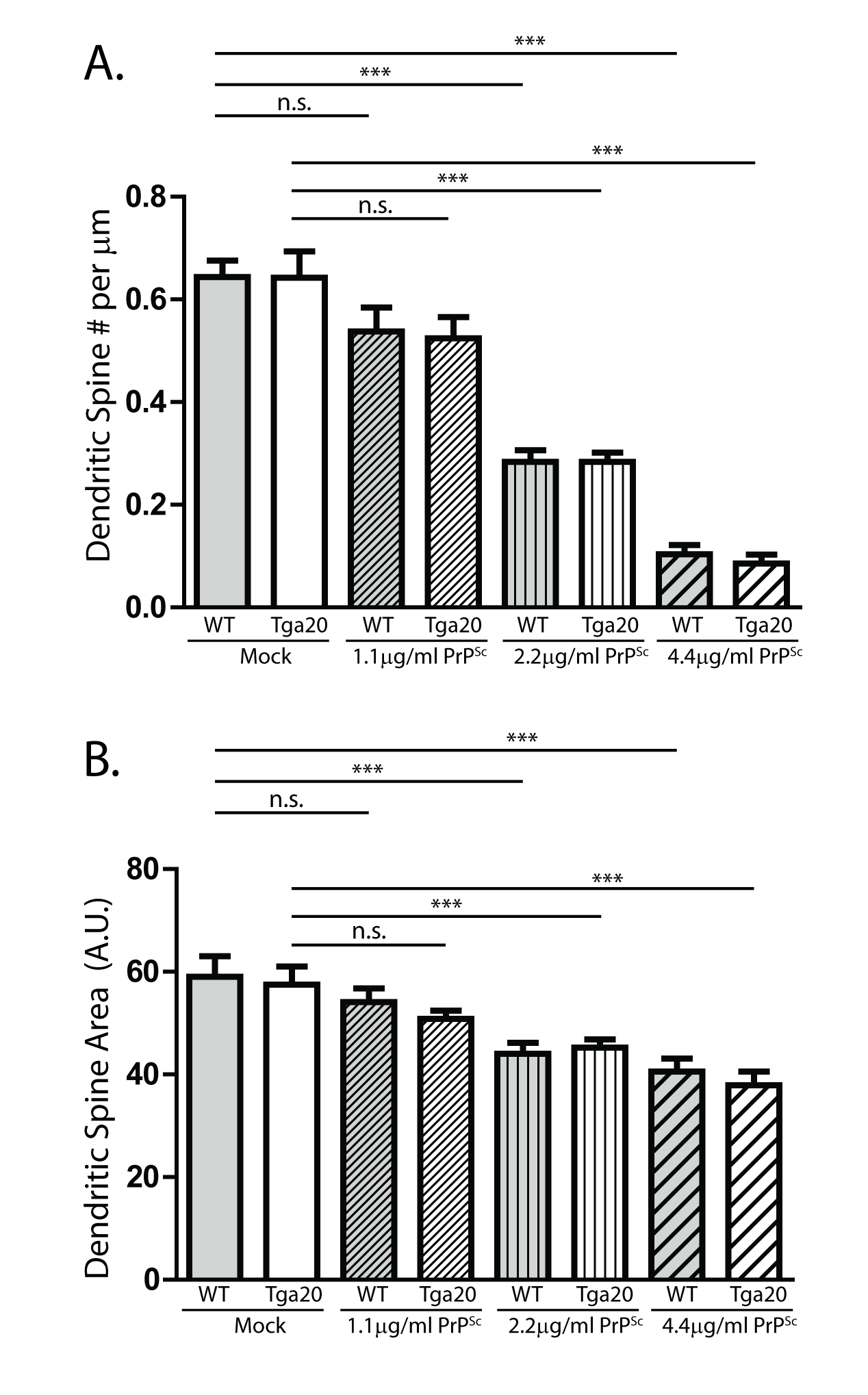

Supplement: S3 Fig — Primary hippocampal neurons from wild-type or Tga20 mice were treated for 24 hr with the indicated concentrations of purified PrPSc (prepared without proteases), or with an equivalent amount of material mock-purified from uninfected brains. Neurons were then fixed and stained with Alexa 488-phalloidin Pooled measurements of spine number (A) and area (B) were collected from 10–15 neurons from 3 independent experiments for each treatment. ***p<0.001 by Student’s t-test; N.S., not significantly different. (TIF) [file ppat.1005623.s003.tif]
